# Hepatitis C in Chronic Kidney Disease After the DAA Revolution: Clinical Decisions, Transplantation, and Implementation Challenges

**DOI:** 10.3390/jcm15176504

**Published:** 2026-08-22

**Authors:** Mostafa Mohrag, Ali Someili, Erwa Elmakki, Mohammed Abdulrasak

**Affiliations:** 1 Department of Internal Medicine, College of Medicine, Jazan University, Jazan 45142, Saudi Arabia; mmohrag@jazanu.edu.sa (M.M.); asomeili@jazanu.edu.sa (A.S.); eelmakki@jazanu.edu.sa (E.E.); 2 Department of Clinical Sciences, Malmö, Lund University, 203 13 Malmo, Sweden; 3 Department of Gastroenterology and Nutrition, Skane University Hospital, 205 02 Malmo, Sweden

**Keywords:** hepatitis C virus, chronic kidney disease, hemodialysis, direct-acting antivirals, kidney transplantation, cryoglobulinemia, glomerulonephritis

## Abstract

Direct-acting antiviral (DAA) therapy has dramatically transformed the management of hepatitis C virus (HCV) infection in chronic kidney disease (CKD). Severe renal impairment and dialysis dependence are no longer considered major barriers to virologic cure. The main difficulties have shifted toward efficient diagnosis, choosing the regimen in the presence of cirrhosis and drug interactions, coordinating treatment with kidney transplantation, managing HCV-associated immune-complex kidney disease, and providing treatment in dialysis and resource-limited settings. This narrative review aims to discuss these issues in a decision-oriented manner, using verified guidelines, systematic reviews, important clinical trials, transplant cohorts, and studies of cryoglobulinemic disease, while explicitly comparing the strength and consistency of the underlying evidence and highlighting areas of genuine clinical uncertainty. Present evidence supports using the recommended DAA regimens without renal dose adjustment, while ribavirin needs dose modification when kidney function is reduced and can result in hemolytic anemia. Kidneys from HCV-viremic donors can be transplanted to recipients without HCV infection when proper informed consent, rapid access to DAA treatment, and organized post-transplant monitoring are available, although the minimum effective duration of peri-transplant antiviral prophylaxis remains unresolved. Antiviral therapy is the first-line treatment for HCV-associated glomerular disease, while rituximab-based immunosuppression is largely reserved for severe, rapidly progressive, or persistent cryoglobulinemic vasculitis. Future progress will depend less on proving antiviral efficacy and more on closing the gaps between screening, confirmatory testing, starting treatment, transplantation pathways, and long-term follow-up, gaps that stem from inequities in diagnostic infrastructure, drug reimbursement, and healthcare-system organization as much as from any remaining biomedical uncertainty.

## 1. Introduction—Changed Clinical Problem

HCV infection and CKD have a bidirectional relationship. HCV can be associated with immune-complex kidney disease and worse outcomes in dialysis patients, while patients receiving hemodialysis have repeated healthcare exposure, which can increase the opportunity of acquiring the infection [1]. Meta-analytic evidence supports an association between HCV infection and incident CKD in the general population [2]. In addition, a large US claims-based cohort found higher risks of CKD, membranoproliferative glomerulonephritis, and cryoglobulinemia among patients with chronic HCV, while HCV treatment was associated with a lower incidence of CKD [3].

The prevalence of HCV among dialysis patients remains much higher than in the general population, although estimates vary across regions and study periods [4]. However, the therapeutic meaning of kidney disease has changed because modern DAA combinations are highly effective in advanced CKD, dialysis, and kidney transplant recipients [5]. Current AASLD-IDSA guidance states that the recommended DAA regimens do not need dose adjustment only because of renal impairment [6]. For this reason, the present review focuses on the clinical decisions that remain difficult after renal impairment is no longer a main barrier for a HCV cure. Because these decisions span diagnosis, drug selection, transplantation timing, and immune-complex kidney disease, they are frequently addressed in separate, specialty-specific studies; this review therefore intentionally integrates evidence across these domains, with the explicit aim of identifying where recommendations are firmly evidence-based, where they rely on limited or indirect evidence, and where genuine clinical controversy remains, rather than restating single-disease guidance in isolation.

## 2. Literature Search Strategy

A targeted narrative search was performed in PubMed/MEDLINE and Google Scholar for publications available through 15 July 2026, with the search restricted to articles published from January 2015 onward to capture the pangenotypic, interferon-free DAA era, while a small number of earlier foundational studies were retained where they remained the primary evidence base for a specific topic (e.g., hemodialysis infection-control recommendations and early rituximab trials for cryoglobulinemic vasculitis). Google Scholar was also used because some relevant articles, online guidance documents, and publications were not indexed in PubMed. The search terms included combinations of “hepatitis C,” “chronic kidney disease,” “renal impairment,” “hemodialysis,” “direct-acting antivirals,” “ribavirin,” “kidney transplantation,” “HCV-viremic donor,” “cryoglobulinemia,” “glomerulonephritis,” and “infection control.” Reference lists of major guidelines, systematic reviews, and pivotal trials were screened as well. Records were included if they reported original clinical data, systematic reviews or meta-analyses, or clinical practice guidelines addressing HCV in the setting of CKD, dialysis, or kidney transplantation, and were available in English; conference abstracts without a peer-reviewed full text, case reports, and studies addressing HCV outside the context of kidney disease were excluded unless they described infection-control principles of direct relevance to dialysis units. This strategy identified 71 unique records, of which the 38 references cited in this review were retained based on guideline status, methodological quality (with preference given to randomized or prospective multicenter studies and systematic reviews when several studies addressed the same question), and direct clinical relevance to the decision points discussed in Section 11. Priority was given to KDIGO and AASLD-IDSA guidance, systematic reviews, randomized or prospective trials, multicenter cohorts, and national registry studies. As this was a narrative review, no protocol was registered, no formal risk-of-bias assessment tool (e.g., ROBIS or AMSTAR-2) was applied, and the selection of studies was not performed by two independent reviewers; these limitations are more consequential given the breadth of clinical domains covered here, and the resulting evidence gaps are made explicit throughout the review, particularly in Section 9.

## 3. Diagnosis: The Main Gap Is Linkage to Care

The CDC recommends at least one lifetime HCV screening test for all adults aged 18 years or more and screening during every pregnancy, except in settings where the prevalence of HCV infection is below 0.1% [7]. A reactive HCV-antibody result means that a previous exposure has occurred, but it cannot by itself differentiate an active infection from a resolved infection or a false-positive result. Due to this, active infection should be confirmed by HCV RNA testing [8]. Reflex HCV RNA testing after a reactive antibody result is useful because it avoids the need for the patient to return for another blood sample, and it is supported in the current HCV guidance [8].

HCV core antigen testing can detect active infection, but its analytical sensitivity is lower than nucleic acid testing and it performs less well when the viral load is low [9]. It can still be useful in places where HCV RNA testing is not available or is expensive, but a negative result should be confirmed by nucleic acid testing when the clinical suspicion remains high [9]. WHO guidance supports simplified HCV care through decentralization, integration, task sharing, point-of-care viral-load testing, and reflex viral-load testing [10]. This is especially relevant in dialysis units, where patients attend several times each week and blood sampling is already being performed, whereby screening, confirmation, pretreatment assessment, and treatment can be connected in one pathway [10].

Before starting treatment, it is important to know if cirrhosis or decompensated liver disease is present, to review previous HCV treatment, and to assess the possible drug–drug interactions [11]. Noninvasive fibrosis assessment is enough for most patients and can determine whether a simplified treatment pathway can be used [11]. KDIGO recommends HCV testing during the initial CKD evaluation, at starting or transferring hemodialysis, and during kidney transplant evaluation, with periodic repeat testing in patients receiving in-center hemodialysis [1]. In patients who cleared HCV, reinfection testing should be done by HCV RNA, as the antibody usually stays reactive [1,8].

## 4. Antiviral Therapy in Advanced CKD and Dialysis and Drivers of Regimen Selection

The evidence for DAA treatment in advanced CKD was established by several trials using both non-sofosbuvir and sofosbuvir-containing regimens [5]. In the C-SURFER trial, 12 weeks of elbasvir–grazoprevir resulted in a high SVR12 rate among patients with genotype 1 infection and CKD stages 4–5, including patients on hemodialysis [12]. The EXPEDITION-4 trial evaluated 12 weeks of glecaprevir–pibrentasvir in patients with HCV genotypes 1–6 and CKD stages 4–5, and it reported SVR12 of 98% by intention-to-treat analysis and 100% by modified intention-to-treat analysis [13]. EXPEDITION-5 included patients with CKD stages 3b-5, where most of them were receiving dialysis, and the overall SVR12 was 97% across the approved treatment durations [14]. These pivotal trials differ meaningfully in design: C-SURFER was a randomized, placebo-controlled, deferred-treatment trial restricted to genotype 1, whereas the EXPEDITION studies were open-label, single-arm trials across multiple genotypes; this distinction, together with other evidence-strength considerations, is discussed further in Section 9.

Sofosbuvir was avoided initially in severe renal impairment because its main metabolite is cleared by the kidney [6]. Later analyses did not show an association between sofosbuvir-based treatment and worsening eGFR or increased risk of end-stage kidney disease in patients who already had renal impairment [15]. A systematic review and meta-analysis also showed high virologic response with sofosbuvir-based regimens in CKD stages 4–5 [16]. Therefore, the current guidance does not require renal dose adjustment for the recommended DAA regimens [6]. Ribavirin is an important exception, as its dose needs adjustment when kidney function is reduced and it may cause clinically significant hemolytic anemia [6,17]. For this reason, hemoglobin should be monitored when ribavirin is used, especially in a patient with CKD or baseline anemia [17].

At present, regimen selection is influenced more by the severity of liver disease, previous DAA exposure, viral genotypes when a non-pangenotypic regimen is used, drug interactions, and the transplant plan, rather than by eGFR alone [6,11]. NS3/4A protease inhibitor-containing regimens, including combinations containing glecaprevir, grazoprevir, or voxilaprevir, are not recommended in patients with decompensated cirrhosis [18].

Kidney transplant recipients need a formal drug-interaction assessment because important interactions can happen between some DAAs and calcineurin inhibitors, particularly cyclosporine [19]. AASLD-IDSA guidance recommends checking the calcineurin inhibitor interaction table before prescribing DAA treatment to a kidney transplant recipient [19]. Medication review should also include statins, colchicine, antiarrhythmics, anticonvulsants, acid-suppressive medications, and herbal products, since these can affect DAA exposure or increase toxicity [11,17].

## 5. Treating HCV Before or After Kidney Transplantation

Treating HCV before kidney transplantation removes the active infection and may make the post-transplant management simpler. On the other hand, delaying treatment may allow the patient to accept a kidney from an HCV-viremic donor [1]. The decision should take into account the severity of liver disease, active extrahepatic manifestations, expected waiting time, donor availability in the local setting, patient preference, and whether post-transplant antiviral treatment is guaranteed [1]. Therefore, this decision cannot be made solely based on kidney function and should be discussed among nephrology, hepatology, infectious diseases, and the transplant team.

DAA therapy remains highly effective after kidney transplantation. In an open-label randomized trial, all kidney transplant recipients with genotype 1 or 4 infection who completed 12 or 24 weeks of ledipasvir–sofosbuvir achieved SVR12 [20]. The MAGELLAN-2 trial also found that 12 weeks of glecaprevir–pibrentasvir was effective and well tolerated in liver or kidney transplant recipients with genotypes 1–6 infection and without cirrhosis [21]. Accordingly, the practical difficulty after transplantation is usually not the antiviral efficacy itself, but avoiding drug interactions, keeping the appropriate immunosuppressant exposure, and having good coordination between the transplant and HCV teams [19,21].

## 6. HCV-Viremic Donor Kidneys

The THINKER pilot trial showed that kidneys from HCV-viremic donors could be transplanted into recipients with no HCV infection and the transmitted infection could then be treated successfully [22]. The EXPANDER trial showed that DAA prophylaxis started immediately before transplantation and continued after it could prevent chronic HCV infection in kidney recipients without HCV infection [23]. In the multicenter MYTHIC study, 30 recipients without HCV received kidneys from HCV-viremic deceased donors and were treated with glecaprevir–pibrentasvir shortly after transplantation, and all of them achieved SVR12 [24].

A systematic review prepared for the KDIGO 2022 guideline included 16 studies with 557 HCV D+/R− kidney recipients and reported an overall SVR12 of 97.7% and one-year allograft survival of 97.6% [25]. National registry data were also reassuring, showing that five-year allograft survival was not worse in recipients of kidneys from HCV-RNA-positive donors compared with recipients of HCV-RNA-negative donor kidneys during the DAA era [26].

However, these good outcomes are dependent on having a reliable system. This should include adequate informed consent, immediate or early access to DAA treatment, virologic monitoring, management of drug interactions, and a plan in case treatment is interrupted or fails [1,27]. Current AASLD-IDSA guidance recommends prophylactic or pre-emptive DAA therapy for recipients without HCV infection receiving non-liver organs from HCV-viremic donors, and very short treatment approaches should not be used outside clinical trials [27]. Therefore, success depends not only on the antiviral regimen but also on the organization of care around transplantation. The optimal minimum duration of peri-transplant DAA prophylaxis, in particular, remains actively debated and is discussed further in Section 9.

## 7. HCV-Associated Glomerular Disease

The most typical renal manifestation of chronic HCV is an immune-complex glomerular disease, usually cryoglobulinemic membranoproliferative glomerulonephritis [1]. KDIGO recommends prompt DAA treatment in HCV-associated glomerular disease, as removing the viral trigger may improve both renal and systemic outcomes [1]. In the RENALCRYOGLOBULINEMIC study, DAA therapy was associated with improved kidney survival and reduced mortality among patients with HCV-associated mixed cryoglobulinemia [28].

In a patient with a typical presentation of HCV-associated immune-complex proliferative glomerulonephritis, antiviral treatment does not need to wait until a kidney biopsy is done [1]. Biopsy remains useful when the presentation is not typical, the diagnosis is uncertain, or kidney function does not improve after virologic cure [1]. Severe cryoglobulinemic vasculitis, rapidly progressive glomerulonephritis, nephrotic syndrome, or life-threatening systemic disease may need immunosuppressive treatment in addition to DAAs [1].

Randomized trials showed that rituximab was effective in severe mixed cryoglobulinemic vasculitis and in HCV-associated cryoglobulinemic vasculitis that did not respond to antiviral therapy [29,30]. Still, the timing and intensity of immunosuppression should be individualized, because advanced CKD, cirrhosis, and concomitant immunosuppression can make the consequences of infection more serious [1].

## 8. Prevention of HCV Transmission in Hemodialysis Units

CDC recommendations for chronic hemodialysis facilities emphasize hand hygiene, changing gloves between patients, aseptic preparation of medications, separation between clean and contaminated areas, and proper environmental disinfection [31]. Mathematical modeling found that adherence to hand hygiene and glove-use recommendations, especially changing gloves between patients, was an important factor affecting HCV transmission risk in hemodialysis centers [32].

A Cochrane review found that the evidence supporting isolation of hemodialysis patients with HCV or using dedicated machines was of very low quality [33]. For this reason, KDIGO recommends strong standard infection-control procedures rather than routine isolation of patients with HCV [1]. In the DAA era, infection control should be connected with treatment, because screening alone will not achieve micro-elimination in a dialysis unit [1,10]. The full care pathway should include scheduled screening, reflex RNA confirmation, pretreatment assessment, starting DAA treatment, documentation of SVR12, and RNA surveillance for patients who continue to have a risk for reinfection [1,8,10].

## 9. Comparative Evidence, Controversies, and Evidence Gaps

The recommendations summarized above are supported by varying levels of evidence, and their certainty therefore varies between topics. The pivotal trials establishing DAA efficacy in advanced CKD differ substantially in design and population: C-SURFER was a randomized, placebo-controlled trial restricted to genotype 1 infection [12], whereas EXPEDITION-4 and EXPEDITION-5 were open-label, single-arm studies across multiple genotypes [13,14]. In addition, reassuring renal safety data for sofosbuvir-based therapy come mainly from observational analyses, while pooled efficacy data are derived from systematic review and meta-analysis [15,16]. Across the pivotal efficacy trials and pooled analyses, virologic response was consistently high [12,13,14,16], while subsequent analyses provided additional reassurance regarding renal safety of sofosbuvir-based therapy [15]. This supports the overall guideline recommendation that renal function alone should not dictate regimen choice [6], but small numeric differences between regimens should not be over-interpreted; regimen selection is more appropriately guided by liver disease severity, drug-interaction profile, genotype coverage when relevant, and transplant logistics.

A more explicit controversy concerns the minimum effective duration of DAA prophylaxis in HCV D+/R− kidney transplantation. Current AASLD-IDSA guidance recommends prophylactic or pre-emptive pangenotypic therapy with 8 weeks of glecaprevir/pibrentasvir when treatment is initiated within the first week after transplantation, or 12 weeks of sofosbuvir/velpatasvir; if initiation of glecaprevir/pibrentasvir is delayed beyond the first week, 12 weeks is recommended [27]. However, attempts to shorten prophylaxis further have produced variable results. In the initial DAPPeR trial, an ultrashort peri-transplant SOF/VEL strategy reduced but did not eliminate donor-derived HCV transmission [34]. Gupta et al. subsequently extended SOF/VEL prophylaxis to 7 days and reported an overall transmission rate of 4%, with transmission occurring in both the SOF/VEL-only and SOF/VEL-plus-ezetimibe groups [35]. In contrast, Feld et al. evaluated one dose of glecaprevir/pibrentasvir plus ezetimibe before transplantation followed by 7 days after transplantation in 30 solid-organ recipients without HCV infection, including 10 kidney recipients, and none developed chronic HCV infection [36]. These findings suggest that the success of abbreviated prophylaxis may depend on both treatment duration and the antiviral regimen used, while the additional contribution of ezetimibe remains uncertain. The minimum reliably effective prophylactic duration therefore remains undefined, and short-duration approaches should remain within clinical trials [27].

The strength of evidence also differs between clinical domains. Evidence supporting HCV-viremic donor kidney transplantation is derived mainly from relatively small prospective single-arm studies, systematic reviews of these cohorts, and registry analyses [22,23,24,25,26]. In contrast, rituximab for severe or refractory cryoglobulinemic vasculitis has been evaluated in randomized controlled trials [29,30], whereas DAA-era evidence regarding renal outcomes in HCV-associated cryoglobulinemia remains mainly observational [28]. These differences should be considered when interpreting the certainty of individual recommendations, and they explain why some areas of current practice are supported by a consistent direction of effect across multiple studies rather than by large head-to-head randomized trials.

Finally, direct comparative-effectiveness data are essentially absent for several practical questions that clinicians actually face including: whether decentralized, dialysis-unit-based HCV treatment achieves comparable cure and reinfection rates to specialist-led care; how to sequence DAA therapy when both transplantation and active cryoglobulinemic disease are present simultaneously; and how outcomes differ when post-transplant DAA access is delayed rather than immediate. These gaps are revisited in Section 12 as explicit priorities for future research.

## 10. Global Implementation and Equity: Barriers to HCV Elimination

In many healthcare settings, the main barriers to HCV elimination are now access to diagnostic testing, fragmented referral pathways, cost of treatment, limited prescribing capacity, and loss of the patient during follow-up, rather than a problem with antiviral efficacy [10,11]. WHO recommends decentralization, integration, and task sharing so that HCV testing and treatment become closer to the patient and fewer patients are lost between diagnosis and treatment [10].

These barriers operate at several distinct levels and rarely act alone. At the diagnostic level, reflex HCV RNA testing and point-of-care viral-load assays remain unavailable or unaffordable in many settings, which can leave active infection under-recognized [9,10]. At the financing level, restrictive reimbursement criteria have historically limited DAA access even in high-income health systems: a systematic evaluation of United States Medicaid policies found that many states restricted sofosbuvir-based treatment according to fibrosis stage, despite broader professional-society recommendations [37]. At the health-system level, the WHO Global Hepatitis Report 2024 shows that HCV diagnosis and treatment coverage remain below the levels needed for elimination by 2030, with important regional inequalities in access to medicines and diagnostics [38]. For dialysis units specifically, resource constraints are compounded by competing clinical priorities, workforce limitations, and the need to coordinate testing, prescribing, and monitoring across nephrology, hepatology, infectious diseases, and pharmacy [1,10]. Consequently, closing the gap between screening and cure requires attention to diagnostic infrastructure, financing, and the internal organization of dialysis programs, and not only to prescribing recommendations.

Dialysis programs can apply these recommendations by adding HCV testing to routine blood sampling, using reflex RNA testing, deciding clearly which team is responsible for starting treatment, and auditing how many RNA-positive patients receive treatment and achieve SVR12 [1,10]. Implementation studies should report the time from diagnosis until treatment, treatment uptake, completion, SVR12, reinfection, and loss to follow-up, and not only report the virologic efficacy of the medication [10].

## 11. Decision-Oriented Framework for Practice

A practical approach starts by confirming active infection with HCV RNA and then assessing cirrhosis, previous HCV treatment, and possible drug interactions [6,8,11]. After this, it should be decided whether kidney transplantation is near, whether an HCV-viremic donor pathway is available, and whether active immune-complex disease needs urgent treatment [1]. In advanced CKD or dialysis, a recommended DAA regimen should be used without reducing the dose only because of CKD, while ribavirin should be adjusted according to renal function and monitored for hemolytic anemia [5,6,12,13,14,15,16,17]. In decompensated cirrhosis, protease inhibitor-containing regimens should be avoided [18].

For a kidney transplant recipient, a formal interaction check and monitoring of the immunosuppressant level are important [19,20,21]. When an HCV-viremic donor kidney is used, the pathway should include informed consent, prophylactic or pre-emptive DAA therapy, and organized HCV RNA monitoring [22,23,24,25,26,27]. In cryoglobulinemic glomerulonephritis, DAA treatment should be started promptly, while rituximab-based therapy can be added in severe or refractory disease [1,28,29,30]. In the dialysis unit, audited standard precautions should be combined with a closed pathway from testing to cure [1,31,32,33]. Finally, HCV RNA should be measured after treatment to document SVR12, while later RNA surveillance should mainly be directed toward patients who remain at risk of reinfection [8,17].

**Figure 1:**
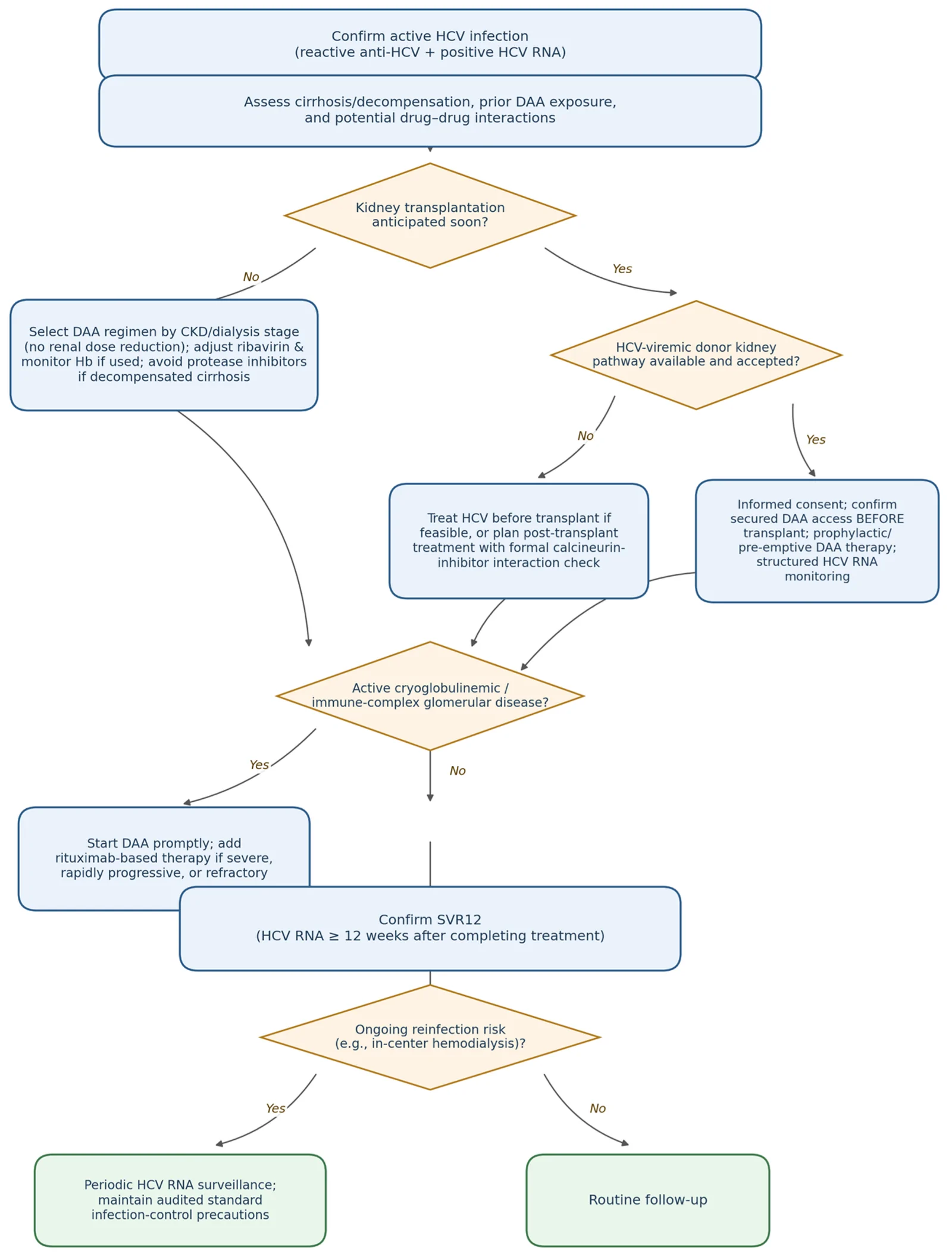
Proposed decision algorithm for the clinical management of hepatitis C virus (HCV) infection in chronic kidney disease (CKD), from confirmation of active infection through transplantation-related and immune-complex glomerular disease decision points to post-treatment surveillance. SVR12: sustained virologic response at 12 weeks; Hb: hemoglobin; DAA: direct-acting antiviral.

Figure 1 summarizes this as a decision algorithm, while Table 1 summarizes the common situations that modify HCV management in CKD. Table 2 provides a summary of the complex clinical scenarios that require personalized judgment.

## 12. Research Priorities

Future research should move beyond studies that only report the cure rate of individual regimens, as the efficacy of recommended DAAs in advanced CKD is already established [5,6]. Important questions include the shortest reliable peri-transplant DAA course, the long-term graft outcomes after HCV D+/R− transplantation, predictors of renal response in cryoglobulinemic disease, retreatment of patients with complex DAA failure, and scalable models in dialysis units that can close the gap between diagnosis and cure [1,25,26,27,28].

Long-term transplant studies should assess not only graft survival, but also kidney function over time, allograft histology, cardiovascular events, malignancy, and patient-reported outcomes [25,26]. There is also a need for implementation studies comparing decentralized care with specialist-led care, using treatment uptake, time to treatment, SVR12, reinfection, and equity as important outcomes [10].

## 13. Conclusions

Severe renal impairment and dialysis are no longer major barriers for HCV cure, because the recommended DAA regimens are highly effective and usually do not need renal dose adjustment [5,6]. Ribavirin remains an exception, whereby its dose needs modification according to renal function and hemoglobin should be monitored because of the risk for hemolytic anemia [6,17]. At present, the main clinical decisions are related to cirrhosis, previous DAA treatment, drug interactions, transplant timing, HCV-viremic donor pathways, and active immune-complex kidney disease [1,11,18,19].

Kidneys from HCV-viremic donors can be transplanted successfully into recipients without HCV infection when prompt DAA treatment and protocolized follow-up are guaranteed [22,23,24,25,26,27]. HCV-associated glomerular disease should be treated promptly with DAAs, while rituximab-based immunosuppression is mainly needed for severe or persistent cryoglobulinemic vasculitis [1,28,29,30]. Future progress will depend on having reliable pathways connecting screening, HCV RNA confirmation, starting treatment, documentation of SVR, reinfection surveillance, and coordination with the transplant team [1,8,10].

## 14. Take-Home Messages

- Recommended DAA regimens generally require no renal dose adjustment across CKD stages, including dialysis; ribavirin is the exception and needs dose modification with hemoglobin monitoring [5,6,17].
- Protease inhibitor-containing regimens should be avoided in decompensated cirrhosis [18], and kidney transplant recipients require formal assessment of DAA–calcineurin inhibitor interactions [19].
- HCV-viremic donor kidneys can be used for recipients without HCV infection when rapid DAA access and structured follow-up are guaranteed; abbreviated prophylactic approaches remain investigational and have produced variable results [22,23,24,25,26,27,34,35,36].
- DAA therapy is first-line for HCV-associated glomerular disease; rituximab is reserved for severe or refractory cryoglobulinemic vasculitis [1,28,29,30].
- HCV elimination in dialysis units requires both audited infection-control practice and a complete pathway from screening to cure, supported by adequate diagnostic infrastructure and equitable treatment access [1,10,31,32,33,37,38].

## Figures and Tables

**Table 1:** Clinical situations that modify HCV management in CKD.

Clinical Situation	Why It Matters	Practical Response	Supporting References
Advanced CKD or dialysis	Kidney function historically restricted therapy, but recommended DAAs now retain high efficacy.	Use a recommended regimen without renal dose reduction; adjust ribavirin when it is required.	[5,6,12,13,14,15,16,17]
Decompensated cirrhosis	NS3/4A protease inhibitor-containing regimens are not recommended.	Use a regimen recommended for decompensated cirrhosis and involve an experienced hepatology/transplant team.	[18]
Kidney transplant recipient	DAAs can interact with calcineurin inhibitors, particularly cyclosporine.	Perform a formal interaction check and monitor immunosuppressant trough levels.	[19,20,21]
HCV-viremic donor kidney	Donor-derived HCV requires prompt and reliable antiviral access.	Use informed consent, prophylactic or pre-emptive DAA therapy, and structured RNA monitoring.	[22,23,24,25,26,27]
Cryoglobulinemic glomerulonephritis	Viral eradication is central, but severe immune-mediated disease may require additional therapy.	Start DAA therapy promptly; add rituximab-based treatment for severe or refractory disease.	[1,28,29,30]
In-center hemodialysis	Nosocomial exposure and reinfection remain possible.	Apply audited standard precautions and maintain a closed testing-to-cure pathway.	[1,31,32,33]

**Table 2:** Complex or uncertain clinical scenarios requiring individualized judgment.

Clinical Scenario	Key Tension	Suggested Approach	Supporting References
Kidney transplantation timing is uncertain (e.g., waiting-list time unknown)	Treating HCV before transplant clears infection but may forgo an HCV-viremic donor offer; deferring treatment preserves that option but leaves infection active.	Base the decision on expected waiting time, local donor-kidney availability, liver disease severity, and patient preference, decided jointly by nephrology, hepatology/infectious diseases, and the transplant team rather than by a fixed rule.	[1,20,21]
Complex polypharmacy with multiple potential drug–drug interactions (e.g., calcineurin inhibitor plus antifungal or antiarrhythmic therapy)	Individual interaction checks may understate cumulative risk when several interacting drugs are combined.	Perform a formal, drug-by-drug interaction review (not only for the calcineurin inhibitor) before DAA initiation; consider temporarily substituting or holding an interacting non-essential medication; increase the frequency of trough-level monitoring during and after DAA therapy.	[11,17,19]
Atypical or uncertain presentation of suspected HCV-associated glomerular disease (e.g., nephrotic-range proteinuria, poor renal response despite SVR12)	Guidance allows DAA initiation without waiting for biopsy in a typical presentation, but atypical features or failure of kidney disease to improve after SVR raise the possibility of an alternative or coexisting diagnosis.	Start DAA therapy without delay when HCV-associated glomerular disease is clinically likely, but perform kidney biopsy when an alternative diagnosis is suspected, kidney disease does not improve or stabilize after SVR, rapidly progressive disease is present, or immunosuppression is being considered.	[1,28,29,30]
HCV-viremic donor kidney offers in a setting where immediate post-transplant DAA access is not guaranteed	Accepting the organ without secured antiviral access risks treatment delay and prolonged recipient viremia, while declining the organ may prolong waiting time.	Confirm antiviral access, including funding or formulary approval and drug supply, before transplantation. If prompt post-transplant DAA therapy cannot be assured, transplantation into a recipient without HCV infection should not proceed within such a protocol.	[1,27,35]

These scenarios presented in Table 2 extend beyond the more straightforward situations summarized in Table 1 and generally warrant multidisciplinary discussion rather than a single fixed rule.

## Data Availability

No new data were created or analyzed in this study. Data sharing is not applicable to this article.
